# Looking on the bright side: biased attention and the human serotonin transporter gene

**DOI:** 10.1098/rspb.2008.1788

**Published:** 2009-02-25

**Authors:** Elaine Fox, Anna Ridgewell, Chris Ashwin

**Affiliations:** Department of Psychology, University of EssexWivenhoe Park, Colchester CO4 3SQ, UK

**Keywords:** cognitive bias, serotonin transporter gene, selective attention, well-being, anxiety, cognitive endophenotype

## Abstract

Humans differ in terms of biased attention for emotional stimuli and these biases can confer differential resilience and vulnerability to emotional disorders. Selective processing of positive emotional information, for example, is associated with enhanced sociability and well-being while a bias for negative material is associated with neuroticism and anxiety. A tendency to selectively *avoid* negative material might also be associated with mental health and well-being. The neurobiological mechanisms underlying these cognitive phenotypes are currently unknown. Here we show for the first time that allelic variation in the promotor region of the serotonin transporter gene (5-HTTLPR) is associated with differential biases for positive and negative affective pictures. Individuals homozygous for the long allele (LL) showed a marked bias to selectively process *positive* affective material alongside selective avoidance of negative affective material. This potentially protective pattern was absent among individuals carrying the short allele (S or SL). Thus, allelic variation on a common genetic polymorphism was associated with the tendency to selectively process positive or negative information. The current study is important in demonstrating a genotype-related alteration in a well-established processing bias, which is a known risk factor in determining both resilience and vulnerability to emotional disorders.

## 1. Introduction

Clinical populations diagnosed with disorders such as depression and anxiety are characterized by a persistent bias to attend towards negative events relative to positive or neutral events (Bar-Haim *et al*. 2007). This research indicates that biased attention for negative emotional stimuli is a significant risk factor in the aetiology and maintenance of abnormal mood states. A causal link is likely in that the experimental induction of biases to attend either towards or away from the negative material in healthy populations results in differential degrees of emotional reactivity to subsequent stressful tasks (MacLeod *et al*. 2002). To date, research has focused on negative biases. However, selective processing of *positive* material, as well as the selective avoidance of negative material, is likely to play an important role in determining mental health and well-being (Fox 1993; Davidson 2004). For example, an inability to experience positive affective states (e.g. optimism, joy) is an important component of serious psychiatric conditions such as depression (Davidson 2004).

Biased attention towards negative or positive material is a useful cognitive endophenotype that is easy to obtain and correlates strongly with normal variations in personality traits that are linked to emotional resilience and vulnerability. While some correlative evidence is available regarding the neural mechanisms that might mediate these biases (Whittle *et al*. 2006), little is known about the precise neurobiological mechanisms that lead to selective processing. While controversial (Abbot 2008), the candidate gene approach indicates that a useful way forwards is to identify genes known to influence biological pathways that are implicated in emotional disorders (Canli *et al*. 2006; Caspi & Moffitt 2006; Canli & Lesch 2007). Instead of assessing variations in genes in relation to a clinical diagnosis such as depression or anxiety, the focus is on intermediate endophenotypes (e.g. self-reported neuroticism, cognitive biases, neural activity) that are known to confer a higher risk of developing a range of emotional disorders. Advocates of this approach have focused on genes that influence serotonin (5-hydroxytryptamine; 5-HT) function in the brain, since variation in 5-HT function is important in modulating mood states and is implicated in depression and anxiety disorders (Heils *et al*. 1996; Hariri & Holmes 2006).

In 1996, a common polymorphism was identified in the promotor region (5-HTTLPR) of the human serotonin transporter gene (5-HTT, SERT, SLC6A4). Carrying the short form of this polymorphism (S allele) modulates the synaptic availability of serotonin (Heils *et al*. 1996), and is also associated with higher levels of self-reported neuroticism (Lesch *et al*. 1996). Subsequent work has shown that the precise effects of the S allele on 5-HT brain function are complex (Hariri & Holmes 2006; Canli & Lesch 2007) and indeed the association with self-reported neuroticism is not always found (Willis-Owen *et al*. 2005). Nevertheless, a 23-year longitudinal study has found that carriers of the S allele are at a higher risk of depression and suicide attempts if they are exposed to major traumatic life events (Caspi *et al*. 2003). The hypothesis that possession of the S allele exaggerates the neurobiological response to stress is supported by animal studies. Rhesus macaques (*Macaca mulatta*) reared in a stressful environment, for example, show increased behavioural and neuroendocrine response to stress if they carry the S allele (Barr *et al*. 2004). Several human studies report increased reactivity of the amygdala in S-allele carriers in response to negative images, such as fearful or angry facial expressions (Hariri *et al*. (2002) see Munafo *et al*. (2008) for recent meta-analysis). Since the amygdala is a central part of the neural circuitry underlying emotional vigilance and arousal (Phelps & LeDoux 2005), this suggests that these neural circuits mediate the association between the S allele of the 5-HTTLPR and emotional reactivity.

While intriguing, functional magnetic resonance imaging studies are difficult to interpret as increased amygdala reactivity is not necessarily a reliable endophenotype to use as a risk factor for heightened stress reactivity. The amygdala and associated circuits are clearly implicated in the fear response (Phelps & LeDoux 2005), but there is also substantive evidence that the amygdala reacts in a more general way to novelty, incongruity and general relevance (Sander *et al*. 2003). Thus, the link between amygdala reactivity and susceptibility to stress is by no means clear. A better endophenotype might be biased attention, which has been found to be reliably associated with increased stress reactivity in a wide range of studies using different methodologies (MacLeod *et al*. 2002; Bar-Haim *et al*. 2007). Moreover, biases both towards and away from particular classes of stimuli differentially predict emotional vulnerability. Thus, a bias to be vigilant for threat-related material is associated with emotional vulnerability, while a bias to selectively *avoid* this type of material is associated with resilience (Fox 1993). Only one study has directly examined biased attention in relation to serotonin transporter genetic variation (Beevers *et al*. 2007). In a small sample of 27 psychiatric inpatients, it was found that carriers of the S allele showed a bias towards anxiety-related words (e.g. scared, attack). While no statistical analyses were reported, examination of their figure 1 indicates that individuals homozygous for the L allele showed a propensity to selectively *avoid* these same stimuli. In the current study, we hypothesize that the S allele is indeed associated with vigilance for threatening material but that those homozygous for the L allele are characterized by selective *avoidance* of threat. Moreover, we also predicted that the LL group would selectively attend to affectively positive material in contrast to those carrying the S allele (SS or SL).

## 2. Material and methods

### (a) Participants

A total of 111 participants underwent a brief interview during which it was established that they had never received any psychiatric diagnosis and they reported that they were taking no medication that affects mental activity. All had normal or corrected-to-normal vision, and gave written informed consent to participate. Standardized questionnaires measuring state and trait anxieties (STAI: Spielberger *et al*. 1983), depressive characteristics (BDI: Beck *et al*. 1996) and the ‘Big 5’ domains of personality, including neuroticism and extroversion (NEO PR-I: Costa & McCrae 1992), were completed. DNA samples (either saliva or eyebrow hair) were obtained from all participants, but genotyping data were available only for 97 of these participants, so data from these 97 are reported.

### (b) Stimuli

**Figure 1:**
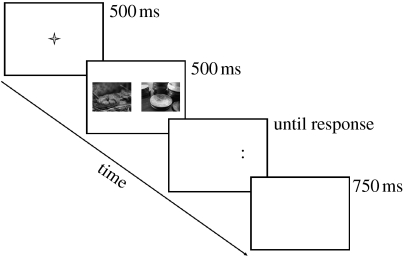
An illustration of a single trial in the dot-probe paradigm. This example shows an affectively negative image (on the left) alongside a neutral image on the right. Affective and neutral images on each trial were matched for subjective arousal level. The target to be responded to appears in the location of the neutral image in this example. The actual images used in the experiment have been replaced with similar public domain images for this illustration to avoid copyright infringement. The images presented here are courtesy of www.photos8.com.

Biased attention was assessed by the dot-probe paradigm that is common in cognitive psychopathology research (Bar-Haim *et al*. (2007) for review). Twenty pictures with a negative valence, 20 with a positive valence and 40 neutral pictures were selected from the *International Affective Picture Set* (Lang *et al*. 2005). All three categories (negative, positive and neutral) were matched on arousal level so that only *valence* differed between any pair of pictures presented. Each trial of the experiment included two of these pictures, one with an emotional valence (either positive or negative) and the other neutral presented on either side of a central fixation point (a cross hair in black Geneva font size 24). All pictures were presented on a white background in grey scale and measured 3.5 cm×4 cm and subtended a visual angle of 6°×8° at a viewing distance of 57 cm. The location of the emotional and neutral pictures was counterbalanced across the experiment. The centre of each picture was 5 cm from fixation, with one picture displayed on the left side of the screen and the other on the right side. Targets consisted of two dots either vertical or horizontal in orientation (: or..) measuring 0.5 cm in length, which appeared in the centre of the location of either the left- or right-hand picture. Both types of target (vertical and horizontal) appeared equally often in the location of the two pictures in each picture pair (left and right side), producing eight different trial combinations for each emotionally valenced picture (see figure 1 for sample trial). As there were 40 emotional pictures, this produced 320 total trials for the experiment, which followed a short set of practice trials.

Participants were tested in a quiet dimly lit testing room, seated approximately 57 cm from a computer monitor placed at eye level. All experimental stimuli were presented on a 17 in. monitor with a resolution of 768×1024 and connected to a Power Macintosh G3 computer running the program Superlab (http://www.superlab.com/) to display stimuli and record responses. A feedback sound was given for any incorrect responses, which were rare in the experiment (the error rate was less than 1%).

### (c) Genotyping

DNA samples were obtained for 111 participants, but extraction of genotyping information was available only for 97 individuals. Genotyping on the serotonin transporter gene was obtained using standard procedures as described in the electronic supplementary material.

## 3. Results

Table 1*a* shows that the three genotype groups did not differ on a range of demographic and self-report measures, and the genotypes were found to be in Hardy–Weinberg equilibrium (*Χ*^2^=0.09). Mean correct reaction time data on the dot-probe task were computed, excluding very fast (less than 150 ms) and very long (more than 2000 ms) reaction times (less than 2% of the data). These mean correct reaction times are shown in table 1*b* and were analysed by means of a 2 (genotyping group: SS, SL, LL)×2 (valence of picture: negative, positive)×2 (location of target: same or different location from affective picture) *analysis of variance*. This analysis revealed that the critical genotyping group×valence of picture×location of target was significant, *F*_2,94_=9.7, *p*<0.001. In order to facilitate the analysis of this interaction, an attentional bias score was computed for both negative and positive images. The *negative bias score* was derived by subtracting the mean reaction times when targets appeared in the location of the negative picture, from the mean reaction times when targets appeared in the location of the neutral picture. Likewise, a *positive bias score* was derived by subtracting the mean reaction times when targets appeared in the location of the positive picture, from the mean reaction times when targets appeared in the location of the neutral picture. Thus a positive value for either the negative or positive indices reflects *vigilance* for the affective picture, whereas a negative value indicates selective avoidance of the affective picture. A score of 0 would indicate no bias either towards or away from the affective picture.

**Figure 2:**
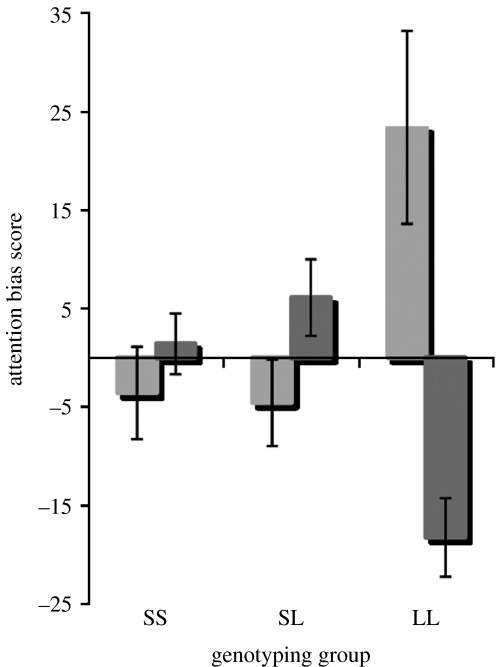
Mean attentional bias scores with standard errors as a function of genotype group and valence of the affective picture. Grey bars indicate bias scores for pictures with a negative valence. Positive scores (above 0) refer to vigilance, negative scores (below 0) refer to avoidance and zero refers to no bias.

The significant genotype (SS, SL, LL)×type of picture (positive, negative) interaction with negative and positive bias scores as the dependent variable is illustrated in figure 2. Follow-up planned comparisons (*t*-tests) were conducted for each genotype group separately and corrected for multiple comparisons by means of the Bonferroni correction (alpha level=0.025). The LL group showed a marked avoidance of negative material (*t*(15)=−4.6, *p*<0.000) alongside a vigilance for positive material (*t*(15)=2.4, *p*<0.015). By contrast, the SS and SL groups showed non-significant tendencies to orient towards negative material (*t*(35)<1 and *t*(44)=1.6, *p*=0.07, respectively) and to selectively avoid positive stimuli (*t*(35)<1 and *t*(44)=1.0, *p*=0.15, respectively). Further analysis revealed that the LL group differed from the other genotype groups in terms of both their avoidance of negative material, as well as their vigilance for positive material (all *p* values<0.001). Moreover, the direction of biased attention for the LL group differed significantly, depending on whether the affective material was negative or positive with negative material eliciting avoidance (mean negative bias=−18.3) and positive images eliciting vigilance (mean positive bias=+23.5; *t*(15)=−3.6, *p*<0.001). These differences did not reach significance for either the SS (mean negative bias=1.4, mean positive bias=−3.6, *t*(35)<1) or the SL groups (mean negative bias=6.1, mean positive bias=−4.6, *t*(45)=1.6, *p*<0.06).

## 4. Discussion

**Figure 3:**
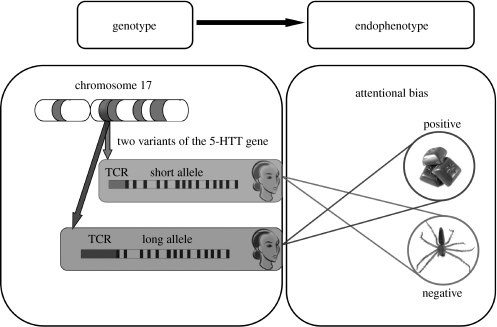
An illustration of how allelic variation in the transcriptional control region (TCR) of the serotonin transporter gene (5-HTT) can influence the nature of selective attention. The affective images presented here are courtesy of www.photos8.com.

The current study investigated whether allelic variation on the 5-HTTLPR gene interacts with biases in attention. This approach attempts to capture gene effects by using dependent measures that are more sensitive than self-report (e.g. neuroticism) or clinical diagnosis (Canli *et al*. 2006; Caspi & Moffitt 2006; Canli & Lesch 2007). Previous studies have used amygdala activation as the endophenotype (Munafo *et al*. (2008) for meta-analysis), while a single study using a measure of biased attention found increased vigilance for threat-related words in S-allele carriers among a psychiatric inpatient group (Beevers *et al*. 2007). The current study is the first to report gene-related variation in biased attention in the healthy population. We found evidence for a strong positive bias in the LL group such that vigilance for positive material was observed among those homozygous for the L allele in addition to a clear avoidance of negative material. This protective pattern was completely absent in S-allele carriers. This result is consistent with a recent report that S-allele carriers and LL groups appear to be affected in opposite ways by life stress. Canli *et al*. (2006) reported that resting activation in the amygdala and hippocampus increased with increasing life stress for SS and SL groups, but *decreased* with increasing life stress in the LL group. Thus, general life stress may induce resilience in some groups (e.g. LL groups), while revealing increased susceptibility to mood disorders in others (e.g. S-allele carriers). As illustrated in figure 3, the current results provide a potential mechanism for this association.

There are a couple of limitations to the current study. First, we did not genotype two relatively new variants that have been identified in the long version of the 5-HTTLPR promotor (L_G_ and L_A_). The L_G_ and the S allele have comparable levels of serotonin transporter expression (Hu *et al*. 2005) and therefore it would be interesting to assess the impact of this variant on the current results. Since the L_G_ is relatively rare (approx. 10%), however, it is unlikely to have influenced the current results to any great extent. Nevertheless, future studies on variation in the 5-HTTLPR and attention should also take into account the additional variants of the L allele. Second, levels of self-reported neuroticism and extraversion did not differ across our genotype groups, which conflicts with some earlier reports (e.g. Lesch *et al*. 1996). However, while early studies did report an association between the S allele and higher levels of neuroticism, several large-scale studies have failed to replicate this association (Willis-Owen *et al*. 2005). The fact that our genotype groups were matched on a range of self-report measures, including neuroticism can be seen as a major strength. If there were differences in these personality traits among the groups, then the biased attention results would be difficult to interpret. As it stands, in spite of no across-group differences in several relevant personality traits (neuroticism, extraversion, depression, state anxiety), we still find clear differences in attentional bias for emotional information across the different genotype groups. This supports our view that differences on the serotonin transporter gene are more likely to predict online measures of information processing as measured here in comparison with more general measures, such as self-report measures of neuroticism. It is concluded that future studies on genetic variation and psychopathology would benefit by obtaining actual measures of selective processing as well as self-report measures. On this point, we also note that we measured biased attention using a standard presentation time of 500 ms. Given that attention is a continuous cognitive process, it is important for future studies to assess the relationship between the 5-HTTLPR and biases in attention at various early and late stages of information processing.

To conclude, the current study found that the presence of positive material induced a strong bias to attend towards this type of material in the LL, but not in either of the SS or SL groups. By contrast, the presence of negative material induced a selective avoidance in the LL group, which was not apparent in S-allele carriers. These low-level biases for affectively salient stimuli play a powerful role in the development of beliefs and general reactivity to significant life events. The current results indicate that a genetically driven tendency to look on the bright side of life is a core cognitive mechanism underlying resilience to general life stress. The absence of this protective bias in S-allele carriers is likely to be linked with the heightened susceptibility to mood disorders such as depression and anxiety that has been reported in this group.

## Figures and Tables

**Table 1:** (*a*) Means and standard deviations (in brackets) for subjective ratings and demographic variables as a function of genotyping group. (*b*) Mean correct reaction times (in milliseconds) and standard deviations (in brackets) on the attention bias (dot-probe) task as a function of genotype group, valence of picture (negative or positive) and location of target (valid, same location as affective picture; invalid, opposite location to affective picture).

	genotype groups		
	
	SS (*n*=36)	SL (*n*=45)	LL (*n*=16)
(*a*)			
trait anxiety	38.8 (8.6)	40.6 (9.8)	39.7 (10.9)
state anxiety at test	31.6 (8.4)	33.7 (8.5)	30.6 (6.8)
depression	7.8 (7.4)	7.6 (7.7)	7.2 (5.0)
neuroticism	16.3 (5.7)	16.5 (4.7)	15.5 (5.6)
extraversion	23.5 (3.3)	23.0 (3.6)	22.0 (3.3)
age	23.6 (7.9)	24.0 (8.1)	25.3 (6.7)
male/female	15/21	27/18	9/7
(*b*)			
picture valence			
negative			
valid	712 (108)	720 (124)	753 (127)
invalid	714 (109)	726 (125)	735 (129)
positive			
valid	703 (106)	708 (123)	732 (137)
invalid	699 (109)	704 (123)	756 (148)

## Supplementary Material

Genotyping Extraction Methods Description of genotyping methodology
