# Supplementary material for: Looking on the bright side: biased attention and the human serotonin transporter gene
Source: Proc Biol Sci. 2009 Feb 25;276(1663):1747–51. doi: 10.1098/rspb.2008.1788 (PMC2674488; doi:10.1098/rspb.2008.1788)
Supplement: Genotyping Extraction Methods — Description of genotyping methodology [file rspb20081788s01.doc]

**Genotyping Methods**

*DNA extraction*

For the majority of participants’ (N = 57) three to four hairs were plucked from the eyebrows with their root ball intact, and these were placed into a labeled 1.5ml Eppendorf tube. These were spun down to ensure the hairs were at the bottom of the tube and then 490μl of 1x Buffer (10mM Tris pH 8.3, 50mM KCL, 0.5% Tween) and 10 μl 10μg/ ml added and the tube was vortexed for 5 seconds. The tube was incubated for 2 hours at 55°C, and then for 15 minutes at 96°C. For the remaining participants (N = 40), DNA samples were taken by running a cotton-tipped swab around the gum line and under the tongue. Saliva was then transferred onto an Indicating FTA Micro Card to which the DNA became inextricably bound.

PCR amplification

Forward (gcaactccctgtacccctccta) and reverse (gtagggtgcaaggagaatgctg) primers were designed in Primer3 (<http://frodo.wi.mit.edu/cgi-bin/primer3/primer3_www.cgi>) to amplify across the 44bp insertion/deletion polymorphism in the serotonin transporter (5-HTT) gene. These were used in a PCR reaction consisting of 1x Hotstart Master Mix (Qiagen) supplemented with 1mM 7-deaza-GTP (Sigma- Aldrich), 0.2μM of each primer and 1μl of the DNA extraction products in a total volume of 25μl. Amplification was carried out on an MJ Tetrad thermal cycler using the following profile: 96°C for 10 minutes followed by 31 cycles of 96°C for 20 seconds, then 60°C for 10 seconds, and finally 72°C for 1 minute.

PCR clean up

PCR products were cleaned up for sequencing by digesting them with 1 unit Exonuclease I (GE healthcare), and 1 unit of Shrimp Alkaline Phosphotase (GE healthcare). These were then subjected to incubation at 37°C for 45 minutes followed by 60°C for 15 minutes.

Sequencing

Reactions were set up containing 1 μl Big DyeTerminator Reaction Mix (Applera), 2μl 5x Dilution Buffer (Applera), 0.2 μM Forward primer and 1μl of cleaned up PCR product in a total volume of 10μl. This reaction was subjected to thermal cycling of 96°C for 10 seconds, 50°C for 5 seconds and 60°C for 4 minute for 25 cycles. Post thermal cycling the reactions were cleaned up using Edge plate (VHBio) according to the manufacturers instructions and subjected to electrophoresis on an ABI3730XL following standard sequencing protocols.

Data analysis

On completion of sequencing the sample files were imported into Sequence Analysis Software (Applera) and the presence or absence of the 44bp insertion/ deletion in the serotonin transporter (5-HTT) gene was determined for each of the individuals screened.
